# Trends in socioeconomic inequalities in stunting prevalence in Latin America and the Caribbean countries: differences between quintiles and deciles

**DOI:** 10.1186/s12939-019-1046-7

**Published:** 2019-10-15

**Authors:** Maria del Pilar Flores-Quispe, María Clara Restrepo-Méndez, Maria Fátima S. Maia, Leonardo Z. Ferreira, Fernando C. Wehrmeister

**Affiliations:** 1 International Center for Equity in Health, Federal University of Pelotas, Rua Marechal Deodoro, 1160 3° Andar, Pelotas, RS 96020-220 Brazil; 2 Post-graduate program in Epidemiology, Federal University of Pelotas, Rua Marechal Deodoro, 1160 3° Andar, Pelotas, RS 96020-220 Brazil; 3 MRC Integrative Epidemiology Unit, Population Health Sciences, University of Bristol, Bristol, UK; 4 Federal University of Rio Grande, Av. Italia, s/n -Km 8-Carreiros, Rio Grande-RS, Brazil

**Keywords:** Child undernutrition, Stunting, Child health, Health equity, Socioeconomic inequality, Latin America countries

## Abstract

**Background:**

With the adoption of the Sustainable Development Goals (SDGs), there is a renewed commitment of tackling the varied challenges of undernutrition, particularly stunting (SDG 2.2). Health equity is also a priority in the SDG agenda and there is an urgent need for disaggregated analyses to identify disadvantaged subgroups. We compared time trends in socioeconomic inequalities obtained through stratification by wealth quintiles and deciles for stunting prevalence.

**Methods:**

We used 37 representative Demographic and Health Surveys and Multiple Indicator Cluster surveys from nine Latin American and Caribbean (LAC) countries conducted between 1996 and 2016. Stunting in children under-5 years was assessed according to the 2006 WHO Child Growth Standards and stratified by wealth quintiles and deciles. Within-country socioeconomic inequalities were measured through concentration index (CIX) and slope index of inequality (SII). We used variance-weighted least squares regression to estimate annual changes.

**Results:**

Eight out of nine countries showed a statistical evidence of reduction in stunting prevalence over time. Differences between extreme deciles were larger than between quintiles in most of countries and at every point in time. However, when using summary measures of inequality, there were no differences in the estimates of SII with the use of deciles and quintiles. In absolute terms, there was a reduction in socioeconomic inequalities in Peru, Honduras, Dominican Republic, Belize, Suriname and Colombia. In relative terms, there was an increase in socioeconomic inequalities in Peru, Bolivia, Haiti, Honduras and Guatemala.

**Conclusions:**

LAC countries have made substantial progress in terms of reducing stunting,. Nevertheless, renewed actions are needed to improve equity. Particularly in those countries were absolute and relative inequalities did not change over time such Bolivia and Guatemala. Finer breakdowns in wealth distribution are expected to elucidate more differences between subgroups; however, this approach is relevant to cast light on those subgroups that are still lagging behind within populations and inform equity-oriented health programs and practices.

## Introduction

The proportion of children under 5 years of age who are stunted is an indicator for the Sustainable Development Goals 2 (SDG 2.2) [1]. Restricted growth as a result of inadequate nutrition and infections is an important cause of morbidity and mortality in children under 5 years [2, 3]. Low educational level, poor access to water and sanitation and health services, as the insufficient household income are some of the determinants of the nutritional state [4]. There have been estimates of trends in national prevalence of stunting, underweight, and wasting by country and by region showing an important reduction in these conditions in children under 5 years in the last decade [5]. Global estimates indicate that the prevalence of stunted children under 5 declined from 40% in 1990 to 24% in 2014 [6, 7]. Economic development, improvements in health systems and progress towards universal coverage have contributed to improved health outcomes for women and children globally [8]. However, the complex interrelation of social, economic, and political determinants of undernutrition results in substantial inequalities between population subgroups [9]. This is especially observed in Latin American countries where health inequalities are still treated as a major concern [10].

The recent establishment of the Sustainable Development Goals and the UN’s labelling of the coming decade as the ‘The Decade of Action on Nutrition’ [7] shows that there is renewed awareness and commitment to tackling the varied challenges of undernutrition, particularly stunting (SDG 2.2) [11]. In addition, the measurement of socioeconomic inequalities in health has received growing attention as the SDG 10 (“reduce inequalities within and among countries”) highlights the importance of reducing disparities to achieve Universal Health Coverage while leaving no one behind, and the SDG 17 calls for disaggregated analyses of targets according to socioeconomic status and other equity stratifiers [11, 12].

The most common way to assess socioeconomic inequalities in health is using quintiles of wealth index [13, 14]. However, in countries where there is still an enormous gap in wealth distribution, finer breakdowns (e.g. deciles) may help identify subgroups that are at higher risk of undernutrition than the rest of the population [12, 15]. Therefore, we aimed to compare time trends in socioeconomic inequalities obtained through stratification by quintiles and deciles of wealth to examine the degree to which trends in socioeconomic inequalities may be underestimated/overestimated by reliance on quintiles.

## Methods

### Study design and data sources

We carried out cross-sectional analyses that relied on data from the Demographic and Health Surveys (DHS) (http://www.dhsprogram.com/) and Multiple Indicator Cluster Surveys (MICS) (http://mics.unicef.org/), both of which are nationally representative household surveys that use the same standard methods to collect data on height measurements. These surveys have been conducted about every 3 to 5 years since the mid-1980s and mid-1990s, respectively. Details on DHS and MICS methodology are published elsewhere [16, 17]. Ethical clearance for the studies were granted in the respective countries and permission to analyze the data was obtained from DHS and MICS.

### Country selection

LAC countries that had at least two surveys available after 1990 with at least 4 years between the earlier and the most recent survey (conducted after 2005) were considered for this study. In addition, these countries must have had information available on anthropometry for children under 5 years. Nine countries met these criteria (see Additional file 1), **Belize, Bolivia, Colombia, Dominican Republic, Guatemala, Haiti, Honduras, Peru and Suriname.** The Guatemala 2008 Reproductive Health Survey (RHS) [18] was used and variables were derived according to DHS and MICS standards. As the last DHS/MICS survey in Guatemala was carried out in 1998 and the most recent was conducted in 2014, we decided to include the RHS survey conducted in 2008 to have intermediate data point between these years. Peru surveys from 2005, 2007 and 2008 were not included due to inconsistencies in the wealth index by quintiles and deciles, which could affect the comparison between the groups of wealth.

### Outcome

Prevalence of stunting was defined as the percent of children, aged 0 to 59 months, whose Z-score of height-for-age is below − 2 standard deviations from the median of the 2006 WHO Child Growth Standards [19].

### Predictors

Prevalence of stunting was disaggregated by household wealth. The wealth index is available in DHS, MICS and RHS surveys and is based on household assets, characteristics of the house, and infrastructure through principal components analyses [14, 20]. The result is a wealth score for each household; then, individuals are ranked according to the total score of the household in which they reside. The sample was then divided into population quintiles and deciles (five and ten groups respectively, with similar sample sizes). By convention, Q1 refers to the 20% poorest and Q5 to the 20% wealthiest households. In similar way, D1 describe the poorest and D10 to the wealthiest.

### Data analyses

#### Measures of inequalities

Two inequality indicators that take the whole distribution of wealth into account were calculated: the slope index of inequality (SII) and the concentration index (CIX). The SII was calculated through a logistic regression model. This approach allows the calculation of the difference in percentage points between the fitted values of the health indicator for the top and the bottom of the wealth distribution [14]. The CIX is based on concept similar to the Gini index. It indicates if a health indicator is concentrated in a particular subgroup. The CIX is expressed on a scale from − 100 to + 100; positive CIX values represent a pro-rich distribution, while negative values indicate that the outcome is concentrated among the poorest groups. The SII expresses absolute inequality, whereas the CIX expresses relative inequality [14, 21]. Both indices were calculated using individual data, where CIX is based on the continuous wealth score and the SII on wealth quantiles.

#### Time-trends

Time-trend analyses of prevalence of stunting and inequality indicators were conducted. Because the time intervals between surveys varied from country to country, average annual changes were calculated to enable standardized comparisons. Variance-weighted least squares regression was used to estimate the average of absolute annual change in prevalence of stunting which allows to consider the different time intervals between surveys, and to test the statistical significance of the observed trends. Survey year was used as the independent variable in the time trend analyses. Annual changes were estimated at the national level and for the poorest (D1 and Q1) and wealthiest (D10 and Q5) deciles and quintiles. Absolute changes are expressed in percentage points per year.

The survey sample design was considered when estimating prevalence of stunting. Analyses were carried out in Stata (StataCorp. 2015. Stata Statistical Software: Release 15. College Station, TX: StataCorp LP).

Table 1 lists the nine countries and their respective surveys included in the analyses.

**Table 1:** Countries selected for the study

Country	Year	Source	National prevalence of stunting (%)	Concentration index	Slope index of inequality	
					Deciles	Quintiles
Belize	2006	MICS	22.5	−29.9	−38.8	− 35.9
Belize	2011	MICS	19.3	−28.4	−32.2	−30.8
Belize	2015	MICS	14.9	−27.7	−24.8	−23.9
Bolivia	1998	DHS	32.9	−23.8	−44.8	− 44.8
Bolivia	2003	DHS	32.3	−24.8	−46.3	−47.0
Bolivia	2008	DHS	27.1	−30.1	−47.1	− 47.0
Colombia	1995	DHS	19.5	−22.6	−25.9	−25.9
Colombia	2000	DHS	18.2	−21.7	−23.2	−22.8
Colombia	2005	DHS	15.7	−25.0	−23.5	−23.1
Colombia	2010	DHS	13.2	−18.6	−14.4	−14.1
Dominican Republic	1996	DHS	13.5	−35.2	−28.5	−28.2
Dominican Republic	2002	DHS	11.4	−26.7	−18.3	− 18.1
Dominican Republic	2007	DHS	9.8	−23.8	−14.0	−13.6
Dominican Republic	2013	DHS	6.9	−24.2	−10.1	−10.0
Guatemala	1995	DHS	55.6	−17.2	−53.4	−53.1
Guatemala	1998	DHS	54.0	−20.1	−60.2	−59.8
Guatemala	2008	RHS	48.0	−22.1	−59.1	−60.9
Guatemala	2014	DHS	46.7	−21.7	−57.4	−57.0
Haiti	1994	DHS	36.8	−15.7	−33.1	−32.6
Haiti	2000	DHS	28.7	−10.8	−30.1	−17.6
Haiti	2005	DHS	29.4	−22.7	− 39.1	−38.6
Haiti	2012	DHS	21.9	−20.8	−26.8	−27.1
Haiti	2016	DHS	21.8	−21.9	−28.8	−29.2
Honduras	2005	DHS	30.0	−30.7	−52.4	−52.5
Honduras	2011	DHS	22.7	−32.3	−42.4	− 42.3
Peru	1996	DHS	31.6	−29.9	−53.6	−53.7
Peru	2000	DHS	31.1	−33.4	− 58.8	−59.6
Peru	2009	DHS	23.8	−36.3	− 49.9	−51.3
Peru	2010	DHS	23.3	−36.5	−49.2	− 49.8
Peru	2011	DHS	19.5	−44.1	−51.3	−51.4
Peru	2012	DHS	18.1	−41.1	−44.6	− 45.0
Peru	2013	DHS	17.7	−44.0	−48.2	−48.7
Peru	2014	DHS	14.8	−43.9	− 41.2	−40.6
Peru	2015	DHS	14.9	−42.0	−39.0	−38.7
Peru	2016	DHS	13.1	−42.3	−35.3	−34.5
Suriname	2006	MICS	10.7	−26.5	−16.9	−16.4
Suriname	2010	MICS	8.8	−21.7	−11.6	−11.2

## Results

Table 2 shows trends in prevalence of stunting at national level and for the poorest (D1 and Q1) and wealthiest (D10 and Q5) deciles and quintiles.

**Table 2:** Trends in national and by wealth stunting prevalence in under five children in LAC countries

Country	Year	National		Deciles				Quintiles			
				D1 (poorest 10%)		D10 (wealthiest 10%)		Q1 (poorest 20%)		Q5 (wealthiest 20%)	
		Prevalence	*P*-value^a^	Prevalence	*P*-value^a^	Prevalence	*P*-value^a^	Prevalence	*P*-value^a^	Prevalence	*P*-value^a^
Belize	2006	22.5		51.0		4.0		38.8		7.8	
Belize	2011	19.3		42.6		9.8		32.9		9.0	
Belize	2015	14.9		34.5		2.1		26.1		5.4	
Annual change		−0.89	< 0.001	−1.87	0.003	−0.30	0.504	−1.36	0.073	−0.74	0.029
Bolivia	1998	32.9		51.8		8.1		49.1		9.0	
Bolivia	2003	32.3		48.5		6.7		48.7		8.7	
Bolivia	2008	27.1		49.0		6.8		45.9		6.5	
Annual change		−0.59	< 0.001	− 0.30	0.254	− 0.12	0.596	− 0.31	0.146	− 0.30	0.090
Colombia	1995	19.5		30.7		10.4		29.5		9.2	
Colombia	2000	18.2		29.9		8.9		26.3		8.9	
Colombia	2005	15.7		28.2		3.5		25.2		4.7	
Colombia	2010	13.2		22.9		7.5		19.4		6.8	
Annual change		−0.44	< 0.001	− 0.55	< 0.001	− 0.08	0.676	− 0.68	< 0.001	− 0.20	0.057
Dominican Republic	1996	13.5		32.0		2.4		26.7		2.9	
Dominican Republic	2002	11.4		22.3		2.5		19.7		3.8	
Dominican Republic	2007	9.8		19.3		5.0		15.8		4.7	
Dominican Republic	2013	6.9		14.2		5.3		11.3		3.9	
Annual change		−0.39	< 0.001	−0.99	< 0.001	0.20	0.135	−0.88	< 0.001	0.09	0.371
Guatemala	1995	55.6		71.5		12.7		70.5		15.8	
Guatemala	1998	54.0		70.0		11.9		71.3		15.0	
Guatemala	2008	48.0		66.9		12.0		67.9		14.3	
Guatemala	2014	46.7		67.7		13.3		66.3		17.4	
Annual change		−0.47	< 0.001	−0.21	0.086	0.052	0.787	−0.23	0.014	0.10	0.369
Haiti	1994	36.8		51.0		10.6		50.1		15.3	
Haiti	2000	28.7		40.8		8.7		37.5		10.1	
Haiti	2005	29.4		46.6		5.6		41.0		8.0	
Haiti	2012	21.9		32.8		5.6		31.0		6.6	
Haiti	2016	21.8		33.8		5.6		33.9		9.1	
Annual change		−0.66	< 0.001	−0.77	< 0.001	−0.20	0.073	−0.69	< 0.001	−0.23	0.009
Honduras	2005	30.0		52.7		4.9		50.4		6.7	
Honduras	2011	22.7		46.5		4.5		42.1		8.0	
Annual change		−1.22	< 0.001	−1.04	0.003	−0.08	0.762	−1.38	< 0.001	0.21	0.325
Peru	1996	31.6		55.1		5.4		52.5		8.4	
Peru	2000	31.1		55.8		3.5		54.1		5.8	
Peru	2009	23.8		47.0		3.5		45.2		4.2	
Peru	2010	23.3		46.3		3.1		44.0		5.4	
Peru	2011	19.5		47.6		1.4		43.6		2.4	
Peru	2012	18.1		42.0		2.5		38.5		3.4	
Peru	2013	17.7		42.4		1.1		38.0		2.9	
Peru	2014	14.8		41.1		3.0		34.2		4.1	
Peru	2015	14.9		37.9		1.8		32.9		2.9	
Peru	2016	13.1		36.9		3.4		30.2		3.6	
Annual change		−1.00	< 0.001	−0.94	< 0.001	−0.13	0.006	−1.20	< 0.001	−0.23	< 0.001
Suriname	2006	10.7		20.7		4.8		16.9		4.4	
Suriname	2010	8.8		17.5		8.0		13.4		5.5	
Annual change		−0.47	0.116	−0.81	0.40	0.79	0.40	−0.85	0.163	0.28	0.622

^a^Variance-weighted least squares regression was used to estimate the average of absolute annual change in prevalence of stunting for each country

Dominican Republic (2013) and Suriname (2010) had the lowest prevalence of stunting (6.9 and 8.8%, respectively), whereas Guatemala (2014) presented the greatest prevalence with almost half of children under five stunted. Eight out of nine countries included showed a strong evidence of reduction in stunting prevalence.

There was evidence of reduction in the prevalence of stunting stratified by deciles in Belize, Colombia, Dominican Republic, Haiti, Honduras and Peru (− 1.9, − 0.6, − 1.0, − 0.8, − 1.0, and − 0.9 percentage points (pp) per year, respectively) among the poorest (D1). Only Peru (− 0.13) had strong evidence of reduction in stunting among the wealthiest (D10).

Similarly, prevalences stratified by quintiles showed that there was a moderate evidence of reduction in stunting in the same five countries above mentioned among the poorest (Q1). Belize and Honduras had the greatest reduction per year (− 1.4 pp). Among the wealthiest (Q5), stunting decreased in Belize (− 0.7 pp. per year), Colombia, Haiti and Peru (− 0.2 pp. per year).

**Fig. 1:**
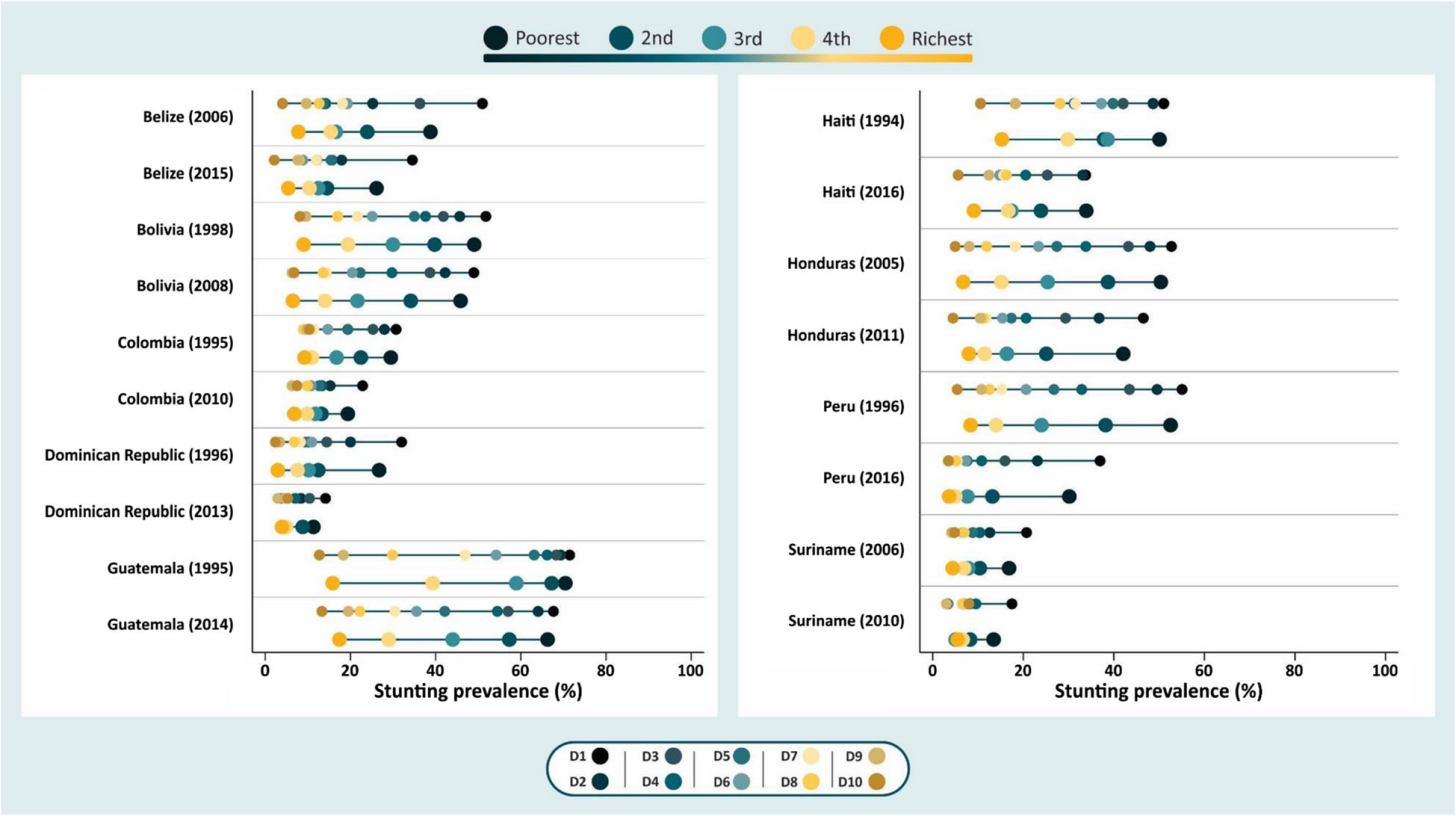
Stunting prevalence for the first and most recent survey in Latin American and Caribbean countries

Figure 1 shows prevalence of stunting by wealth deciles and quintiles for the baseline and the most recent survey of each country. Each survey presents a line for deciles and one for quintiles.

In Belize and Dominican Republic, the poorest 10% (D1) has a prevalence of stunting 1.3 and 1.2 times (respectively) higher than the poorest 20% (Q1), and these differences have persisted over time. Among the wealthiest, the greatest differences between D10 and Q5 in stunting prevalence were found in Belize, Guatemala and Haiti. These differences decreased over the years in four out of nine selected countries.

**Fig. 2:**
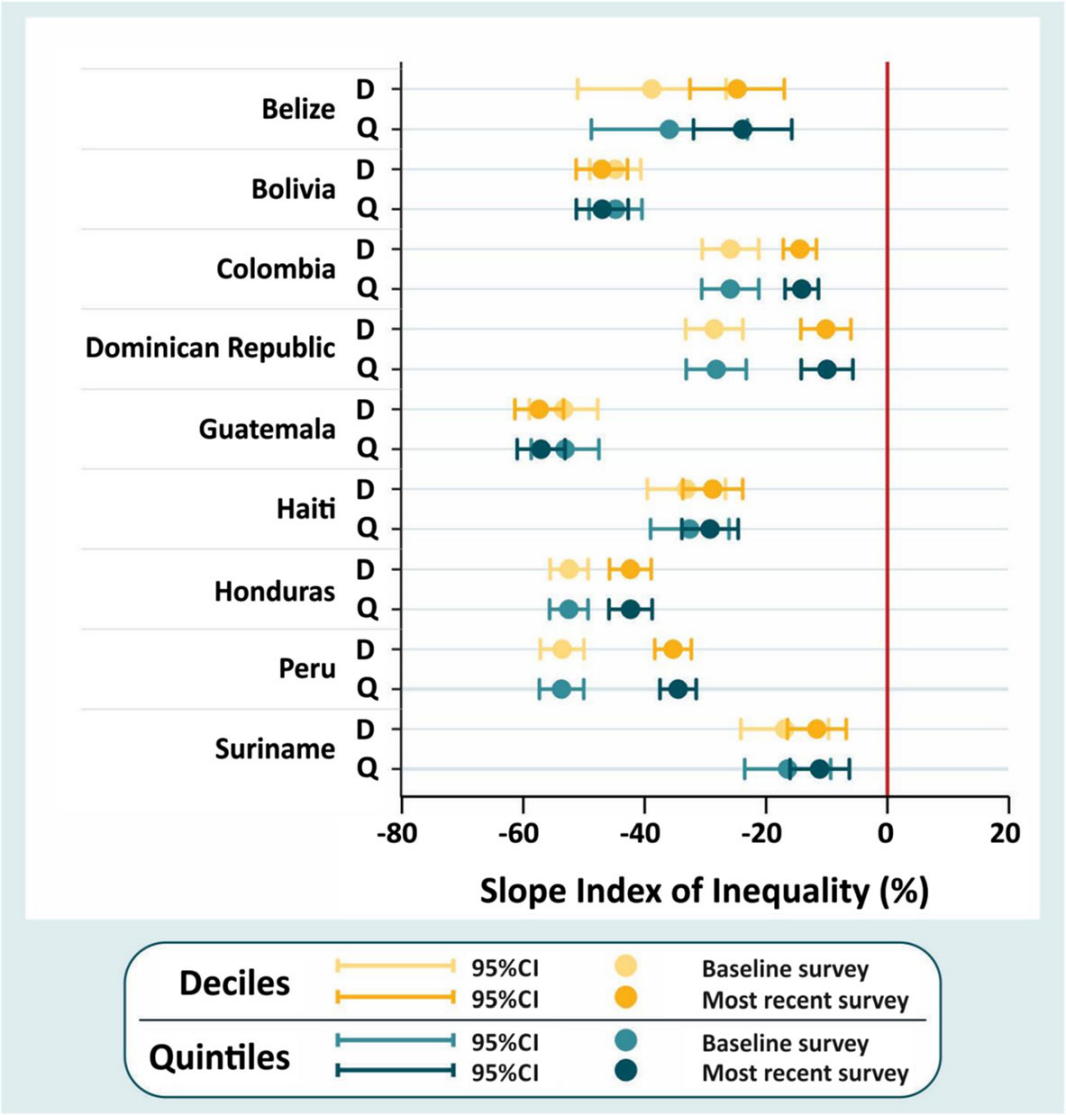
Changes in absolute inequalities by wealth quintiles and deciles in stunting prevalence in LAC countries

Figure 2 show trends in absolute (SII) socioeconomic inequalities between the baseline and the most recent survey for each country, where zero indicates equality. There were no differences in the estimates of SII with the use of deciles and quintiles. In absolute terms, there was evidence of reduction in socioeconomic inequalities in Peru, Honduras, Dominican Republic and Colombia (Fig. 2).

**Fig. 3:**
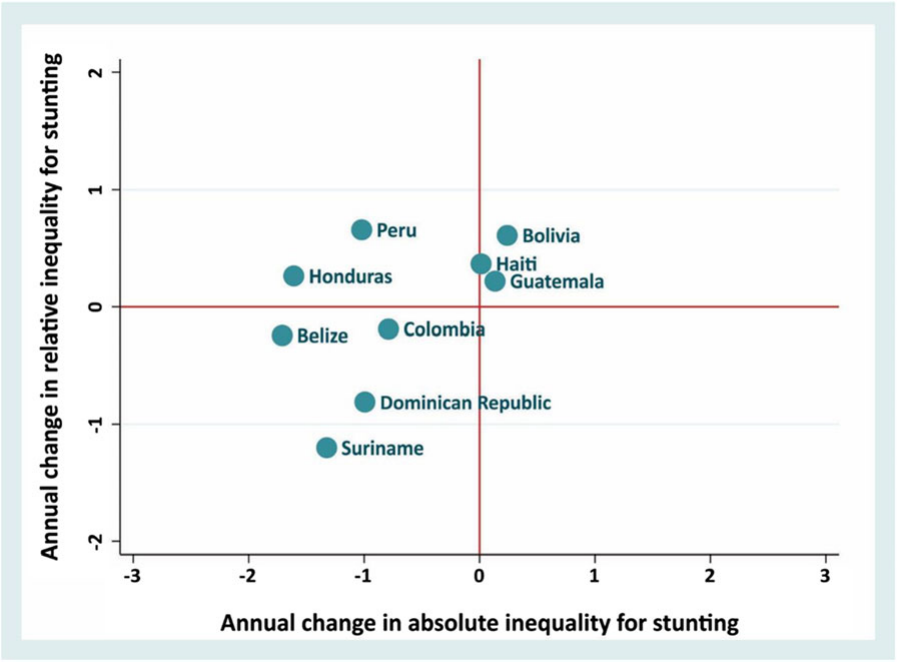
Average annual change in absolute versus relative inequalities in stunting prevalence in LAC countries

Figure 3 shows the performance of countries in relation to reduction of both absolute and relative inequalities over time. Belize, Suriname, Colombia and Dominican Republic, which are located in the lower left quadrant, achieved the best progress in reduction of inequalities, whereas Bolivia and Guatemala showed an increase in absolute and relative inequalities. Additional file 1 shows the annual change in Concentration Index and Slope Index of Inequality for stunting for each country.

## Discussion

Our results suggest a reduction in the prevalence of stunting in most of countries, particularly among the poorest, independently the use of deciles or quintiles. In addition, socioeconomic inequalities in stunting prevalence seem to be decreasing in most of countries in at least one measurement (absolute or relative).

As documented in previous studies, the reduction in the global prevalence of stunting may be a result of different policies implemented by some low- and middle-income countries that have improved purchasing power in low-income families, female education, basic sanitation services and health care [5, 22–24]. In particularly those interventions in the health and other sectors including conditional cash transfers (CCT) and universal health-care coverage may have played an important role in increasing access to health among more vulnerable population [8] and, therefore, in reducing stunting in children under 5 years in Latin American and Caribbean countries [25]. In our analyses, 8 out of 9 countries had a evidence of reduction in prevalence of stunting. Greater annual decreases were observed in Honduras, Peru, Belize and Haiti. However, it is worth noting that only in Dominican Republic and Suriname less than 10% of children under five were stunted when considering the most recent survey. In their baseline surveys the national prevalence was already less than 15%. These countries have the greater proportion of their population living in urban areas unlike the other countries [26, 27]. Also, some actions were implemented in Dominican Republic that could have contributed with the decrease of stunting prevalence, like the General Health Law and the Social Security System, approved in 2001 [26], and the conditional cash transfer program “Solidarity” which was created in 2005 [28]. In Suriname, the Regional Health Services provides preventive and health care interventions focused on under-five children since 1991 [27]. These policies might have contributed with the reduction of e stunting prevalence, particularly in the poorest group, where the decrease was nearly 1 % point per year in both countries. In addition, the richest quintile did not change over time in both countries, which was also reflected in the reduction of inequalities in stunting prevalence. However, further studies are needed to explore underlying reasons that led to these changes. It is beyond the scope of this study to explore such reasons.

Previous studies have reported similar results showing a reduction in the prevalence of stunting in Latin America region from 23.7% in 1990 to 13.5% in 2010 [22, 29, 30], higher to the ones observed for Africa and South Asia regions [31].

Most of studies that previously assessed trends in inequalities in stunting used quintiles of wealth index to show differences in stunting prevalence among socioeconomic groups and to highlight under-served groups that are being left behind and not reached by health interventions. The use of the wealth index divided into quintiles for the evaluation of health-related outcomes is well-known and accepted in the scientific literature [13, 30]. Its use is due, among other things, to its easy understanding and for statistical power issues, avoiding groups of very small sample size [21]. However, in surveys of national representativeness with large samples, the assessment by deciles may show more pronounced differences between the socioeconomic subgroups when comparing wealth deciles and quintiles, the last could hide important differences. Finer disaggregation could help policy makers to identify the target population for a given intervention [32].

When comparing progress of countries in reducing prevalence and inequalities over time, we need to interpret results with caution as the higher the initial prevalence, easily it decreases over time if appropriate interventions are implemented, as it has been described previously [12]. For instance, our analyses show that the absolute reduction in the prevalence of stunting over time was larger between the poorest 10% (D1) than the wealthiest 10% (D10) in all countries. Similar results were observed when assessing prevalence by wealth quintiles. Traditionally the poorest groups present higher prevalence of stunting.

Among the countries that reduced the prevalence of stunting in D1, the initial prevalences varied between 32 and 55%. Despite these advances, the differences with the wealthiest (D10) were still marked, as the case of Guatemala, Honduras, and Bolivia which showed the greatest differences (more than 40 pp). The same comparison was observed in these countries when comparing Q1 and Q5. These countries have implemented some strategies to promote family and community health care. In Peru, in 2009, the law of Universal Health Insurance was expanded for all citizens and in 2011 the Comprehensive Family and Community Care model was introduced with the aim of preventing and promoting health, especially addressing the poorest and most remote populations [8]. In the case of Honduras, since 1990, it has the Family Allowance Programme and in 2009 the Health Services Decentralization Plan was launched with the objective of promoting, prevention and community familiar care [28, 33]. However, there are no studies that evaluate the impact of these actions on the reduction and socioeconomic inequality of stunting prevalence.

Despite the usefulness of the deciles to evaluate more extreme groups within the distribution of wealth, using them to calculate complex measures of relative (CIX) or absolute (SII) inequality do not appear to be much different than the same measures using wealth quintiles. This was also found in a previous study [15].

Guatemala and Bolivia reported the highest prevalence of stunting in their most recent survey and an increased in absolute and relative inequalities. These countries have the highest proportion of household in rural areas (46 and 37% respectively), five to more members per household, and less expenditure on food when compared to other countries in the region [34]. In 2008 the conditional cash transfer program “Mi Familia Progresa” was created in Guatemala, and a similar programme called “Juancito Pinto Grant” began in Bolivia in 2006 [28]. The late start of these programmes when compared to other programmes in other countries of the region might explain the little progress in the reduction of stunting over time. However, stunting is driven by a complex net of determinants including social, economic, political and cultural factors [5]. For instance, the intricate relationship between ethnicity and poverty [35] could also play an important role on stunting prevalence in Bolivia and Guatemala. Both countries have a high proportion of indigenous populations and a recent study showed slow improvement on stunting prevalence over time in Guatemala among indigenous population [36]. Also, a report from Food and Agriculture Organization, launched in 2018, showed that Bolivia and Guatemala belong to the most vulnerable countries in terms of food insecurity [37]. Therefore, more studies are needed to understand better the social, political, health-related and other contextual factors that may explain little progress in some countries and successful stories in others.

On the other hand, Colombia is one of the countries that in addition to reduce the prevalence of stunting (with initial prevalence of 20%), also managed to reduce absolute and relative inequalities. Since 1993, this country has implemented a series of programs (Obligatory Health Plan, Families in Action) as part of the reform of the health system, as well as its decentralization, with the aim that health benefits reach disadvantaged populations, which might have contributed to increase universal health coverage [8]. An analysis from Colombian surveys showed an important improvements on several nutritional indicators in the period 2000 to 2010 [38].

Our findings should be interpreted in the light of some limitations. As we have no surveys available from all Latin American and Caribbean countries, we cannot emphatically conclude on the progress achieved by this region in reducing stunting inequalities. However, based on the 9 countries studied, these results suggest that some Latin American and Caribbean countries have managed to make marked progress in reducing stunting prevalence at the national level, as well as reduce the gap between rich and poor. The experience of these countries can serve as examples for other Latin American and Caribbean countries, although further research is required to understand which political, social, economic, and health-related factors account for the persisting inequities in some countries and substantial reduction in others.

Another limitation is the lack of more recent surveys for some countries such as Bolivia, whose most recent DHS survey is from more than 10 years ago, which may not reflect the current stunting prevalence in that country. In addition, the division of the wealth index by deciles may reduce the sample size of some groups. For instance, in Belize (2006), the 10th decile (D10) had fewer than 40 children which can affect the precision of our estimates. In most cases, the sample size of deciles was always around or greater than 100.

Among the strengths, we can highlight the standardization of anthropometric measurements, which allows a good degree of comparability between stunting estimates. In addition, the national representativeness of surveys also allows to make inferences about the situation of stunting prevalence in each country.

## Conclusion

Substantial reductions in the national prevalence of stunting can be observed in Latin America and Caribbean, however, these trends do not occur in a similar way among the wealth groups. These differences are more marked when using wealth deciles. However, the use of deciles and quintiles for estimating complex measures of inequalities do not appear to be affect conclusions in relation to trends in socioeconomic inequalities as there is no difference in the results obtained.

The stratification by wealth deciles may be important to show inequalities even more striking and highlighting the need for public policies focused in the most vulnerable groups as an important strategy to cope with chronic malnutrition. We hope that these findings will inform policy debates on strategies to reduce health inequalities in Latin American region.

## Supplementary information

**Additional file 1.** Annual change in absolute and relative inequalities for stunting in LAC countries.

## Data Availability

Data supporting our findings can be found in the following links: • DHS surveys: http://dhsprogram.com/ • MICS surveys: http://mics.unicef.org/ • RHS surveys: http://ghdx.healthdata.org/series/reproductive-health-survey-rhs/

## Abbreviations

CCT: Conditional cash transfers
CIX: Concentration index
DHS: Demographic Health Surveys
LAC: Latin American and Caribbean
MICS: Multiple Indicator Cluster Surveys
RHS: Reproductive Health Survey
SDGs: Sustainable Development Goals
SII: Slope index of inequality
WHO: World Health Organization
