# Supplementary material for: Trends in socioeconomic inequalities in stunting prevalence in Latin America and the Caribbean countries: differences between quintiles and deciles
Source: Int J Equity Health. 2019 Oct 15;18:156. doi: 10.1186/s12939-019-1046-7 (PMC6794733; doi:10.1186/s12939-019-1046-7)
Supplement: Supplementary file 1 — Additional file 1. Annual change in absolute and relative inequalities for stunting in LAC countries. [file 12939_2019_1046_MOESM1_ESM.pdf]

Additional file 1. Annual change in absolute and relative inequalities for stunting in LAC countries

Country	Concentration Index annual change	IC 95%	Slope Index of Inequality annual change	IC 95%
Belize	-1.71	-3.31;-0.11	-0.24	-1.43;0.94
Bolivia	0.24	-0.36;0.84	0.61	0.26;0.96
Colombia	-0.79	-1.12;-0.46	-0.19	-0.51;0.13
Dominican Republic	-0.99	-1.34;-0.65	-0.81	-1.35;-0.27
Guatemala	0.13	-0.20;0.47	0.22	0.09;0.35
Haiti	0.01	-0.30;0.32	0.37	0.18;0.55
Honduras	-1.61	-2.38;-0.84	0.26	-0.25;0.78
Peru	-1.02	-1.19;-0.86	0.66	0.55;0.77
Suriname	-1.32	-3.50;0.85	-1.2	-4.55;2.15
